# Penicillin acylase-catalyzed synthesis of *N*-bromoacetyl-7-aminocephalosporanic acid, the key intermediate for the production of cefathiamidine

**DOI:** 10.1186/s40643-016-0127-3

**Published:** 2016-11-19

**Authors:** Xiao-Li Zhang, Min-Hua Zong, Ning Li

**Affiliations:** School of Food Science and Engineering, South China University of Technology, 381 Wushan Road, Guangzhou, 510640 China

**Keywords:** 7-Aminocephalosporanic acid, Enzyme catalysis, Cephalosporins, Penicillin acylase, Semi-synthetic antibiotics

## Abstract

**Background:**

Enzymatic approaches have become promising alternatives to chemical methods for the production of semi-synthetic β-lactam antibiotics. In this work, enzymatic synthesis of *N*-bromoacetyl-7-aminocephalosporanic acid (*N*-bromoacetyl-7-ACA), the key intermediate for the production of cefathiamidine, was reported for the first time.

**Results:**

Of the immobilized penicillin acylases (PAs) tested, PGA-750 was the best biocatalyst. Optimization of the biocatalytic process was conducted. The optimal acyl donor, molar ratio of acyl donor to 7-ACA, pH, temperature, 7-ACA concentration, and enzyme dosage were methyl bromoacetate, 3, 7.5, 20 °C, 50 mmol/L and 4 U/mL, respectively. Under the optimal conditions, enzymatic *N*-acylation of 7-ACA with methyl bromoacetate afforded the desired product with the yield of 85% in 2 h, where the synthesis/hydrolysis (S/H) ratio was approximately 1.5. The immobilized enzyme PGA-750 exhibited good operational stability, and the relative yields of approximately 90% and 63% were achieved, respectively, when it was reused in 7th and 11th batch.

**Conclusions:**

An enzymatic approach to *N*-bromoacetyl-7-ACA, the key intermediate for the industrial production of cefathiamidine, has been developed successfully in a fully aqueous medium. The present work may open up a novel opportunity for the production of cefathiamidine through a simple and green process.

**Graphical abstract:**
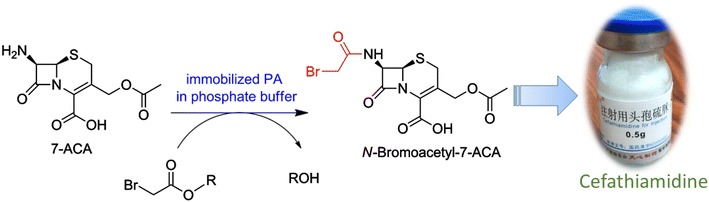
Enzymatic synthesis of *N*-bromoacetyl-7-ACA, the key intermediate for the production of cefathiamidine, was reported for the first time.

**Electronic supplementary material:**

The online version of this article (doi:10.1186/s40643-016-0127-3) contains supplementary material, which is available to authorized users.

## Background

**Fig. 1:**
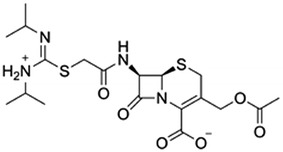
Structure of cefathiamidine

Cefathiamidine was one of the first-generation semi-synthetic cephalosporins, which was firstly synthesized by Wang et al. in 1974 and reported in 1978 (Wang et al. 1978). It is an internal salt of 7-(α-((*N*,*N*′-diisopropylamidino)thio)acetylamino)cephalosporanic acid (Fig. 1). Cefathiamidine showed good antimicrobial activities against both Gram-negative and Gram-positive bacteria; in particular, this semi-synthetic cephalosporin was highly effective for in vitro killing enterococci (Chen and Williams 1983; Dai et al. 1978). Presently, it remains one of the important clinic drugs for the treatment and prevention of various bacterial infections in China (Li 2003; Yang et al. 2006; Li et al. 2016). Currently, cefathiamidine is industrially produced through two-step cascade chemical transformations (Wang et al. 1978; Wang and Li 2010). Briefly, 7-aminocephalosporanic acid (7-ACA) was subjected to *N*-bromoacetylation with highly active bromoacetyl bromide under <5 °C in alkaline media, furnishing *N*-bromoacetyl-7-ACA. Then, the condensation of *N*-bromoacetyl-7-ACA and *N*,*N*′-diisopropylthiourea in CH_2_Cl_2_ gave the desired product cefathiamidine. Although various chemical approaches to cefathiamidine were developed (Liu et al. 2001; Fu and Tian 2009), they were associated with many problems such as requiring low temperature, using toxic chemicals, organic solvents and environmentally unfriendly catalysts. In addition, the highest yield of chemical synthesis of *N*-bromoacetyl-7-ACA was approximately 73% (Wang and Li 2010).

**Scheme 1:**
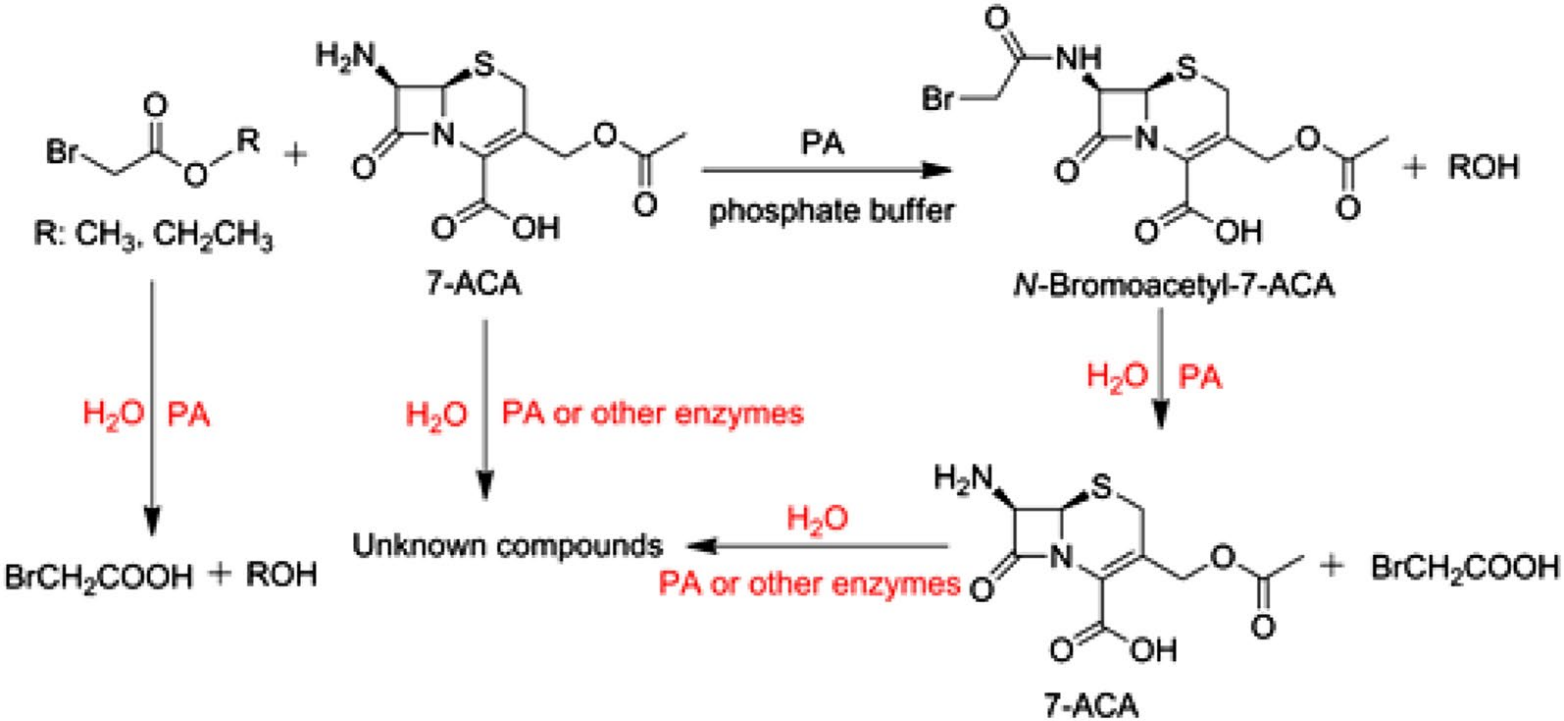
Kinetically controlled enzymatic synthesis of *N*-bromoacetyl-7-ACA

Penicillin acylases (PAs, also known as either penicillin amidase or penicillin amidohydrolase, EC 3.5.1.11) are robust industrial biocatalysts. For example, PAs have been used widely for several decades for the industrial production of β-lactam nuclei—6-aminopenicillanic acid (6-APA) and 7-amino-3-deacetoxycephalosporanic acid (7-ADCA) via hydrolysis of penicillin G and cephalosporin G, respectively (Arroyo et al. 2003; Wegman et al. 2001). In addition, PAs have proven to be excellent catalysts for the *N*-acylation of β-lactam nuclei via thermodynamically or kinetically controlled strategies, which affords semi-synthetic β-lactam antibiotics such as ampicillin, amoxicillin, cefazolin, and cephalexin (Wegman et al. 2001; Volpato et al. 2010; Srirangan et al. 2013). Currently, the large-scale enzymatic production of many semi-synthetic β-lactam antibiotics such as cephalexin has been realized with PAs as biocatalysts (Wegman et al. 2001; Bruggink et al. 1998). Compared to chemical methods, the enzymatic routes appear to be preferable for the synthesis of semi-synthetic β-lactam antibiotics because of many advantages such as mild reaction conditions, avoiding use of toxic chemicals, producing less waste, and being simple and environmentally friendly (Arroyo et al. 2003; Bruggink et al. 1998; Marešová et al. 2014). However, to our knowledge, enzymatic or chemoenzymatic synthesis of cefathiamidine has not been reported yet. As described above, *N*-bromoacetyl-7-ACA is the key intermediate for the synthesis of cefathiamidine. In this work, therefore, kinetically controlled enzymatic synthesis of *N*-bromoacetyl-7-ACA from 7-ACA and bromoacetates was reported for the first time (Scheme 1). In addition to the synthesis reaction, PA-catalyzed side reactions including the hydrolysis of acyl donor and the desired product occurred simultaneously, affording bromoacetic acid and alcohols as the by-products. In addition, unknown compounds were also produced from 7-ACA in the presence of the immobilized enzyme.

## Methods

### Materials

PA from *Bacillus megaterium* (PGA-750) covalently immobilized on polymethacrylate resin was purchased from Shunfeng Haider Co. Ltd. (Zhejiang, China). PAs from *Escherichia coli* (PGA-II) and from *Achromobacter* sp. (PGA-IV) immobilized on polyacrylate resin were kindly donated by Shijiazhuang Pharmaceutical Group Co. Ltd. (Hebei, China). PAs (SIPA-III, SIPA-IV, and SIPA-V) from *E. coli* immobilized on polystyrene resin were purchased from Hunan Flag Biotech Co. Ltd. (Hunan, China). All the PAs used in this work were penicillin G acylases. 7-ACA and *N*-bromoacetyl-7-ACA were kindly provided by Baiyunshan Chemical Pharmaceutical Factory (Guangzhou, China). Methyl bromoacetate, ethyl bromoacetate, penicillin G potassium salt (PGK), and *p*-dimethylaminobenzaldehyde (PDAB) were obtained from Macklin Biochemical Co. Ltd. (Shanghai, China). 6-APA was purchased from Aladdin Industrial Co. (Shanghai, China). All other reagents were of analytical grade and obtained from commercial sources.

### Enzyme activity assay

The enzyme activities of various immobilized PAs were determined according to the previous methods (Ferreira et al. 2007; Shewale et al. 1987), with some modifications. Briefly, the immobilized enzyme was added into 5 mL of phosphate buffer (100 mmol/L, pH 7.5) containing PGK (1.08 mmol, 216 mmol/L). After the mixture was incubated at 37 °C and 200 r/min for 10 min, 5 mL ethanol was added to quench the enzymatic reaction. Then 1 mL of sample that was withdrawn from the mixture was mixed with 2 mL of acetic acid-NaOH buffer (being composed of 2 mL of 20% acetic acid and 1 mL of 0.05 mol/L NaOH), followed by adding 1 mL of 5% PDAB dissolved in anhydrous methanol. After incubation at 37 °C for 20 min, the absorbance was measured spectrophotometrically at 415 nm. The controls without enzyme were run simultaneously. 1 U was defined as the amount of enzyme that released 1 μmol 6-APA per minute at pH 7.5 and 37 °C. The specific activities of SIPA-III, SIPA-IV, SIPA-V, PGA-II, PGA-IV, and PGA-750 were 98.8, 44.9, 72.5, 78.9, 22.7, and 624.5 U/g, respectively.

### Enzymatic synthesis of *N*-bromoacetyl-7-ACA

Typically, immobilized PA (3 U/mL) was added to 10 mL of phosphate buffer (100 mmol/L, pH 7.5) containing 7-ACA (40 mmol/L) and methyl bromoacetate (120 mmol/L), and then the mixture was incubated at 20 °C and 200 r/min. Aliquots were withdrawn from the reaction mixture at specified time intervals and diluted with the corresponding mobile phase prior to HPLC analysis. The initial reaction rate (*V*_0_) was calculated based on the decrease in the 7-ACA concentrations at the initial reaction stage (generally within 5 min).

The conversion was calculated according to the following equation: ${\text{Conversion (\% ) = }}\frac{{C{\text{s}} - 0 - C{\text{s}} - {\text{t}}}}{{C{\text{s}} - 0}}{ \times }100$, where *C*_s−0_ and *C*_s−t_ are the 7-ACA concentrations at 0 and t h, respectively.

The yield was calculated according to the following equation: ${\text{Yield (\% ) = }}\frac{{C{\text{p}} - {\text{t}}}}{{C{\text{s}} - 0}}{ \times }100$, where *C*_s−0_ and *C*_p−t_ are the 7-ACA concentration at 0 h and the *N*-bromoacetyl-7-ACA concentration at t h, respectively.

The synthesis/hydrolysis (S/H) ratio was calculated according to the following equation: ${\text{S/H ratio = }}\frac{{C{\text{p}} - {\text{t}}}}{{C{\text{ester}} - 0- C{\text{ester}} - {\text{t}} - C{\text{p}}\text{ - }{\text{t}}}}$, where *C*_p−t_, *C*_ester−0_, and *C*_ester−t_ are the *N*-bromoacetyl-7-ACA concentration at t h, the bromoacetate concentrations at 0 and t h, respectively. All the experiments were carried out at least in duplicate, and all the results were expressed as the means ± standard deviations.

### Reuse of the immobilized enzyme PGA-750

PGA-750 (40 U) was added to 10 mL of phosphate buffer (100 mmol/L, pH 7.5) containing 7-ACA (50 mmol/L) and methyl bromoacetate (150 mmol/L), and then the mixture was incubated at 20 °C and 200 r/min for 2 h. Then, the immobilized enzyme was filtered out and washed three times with 10 mL of phosphate buffer (100 mmol/L, pH 7.5). The isolated enzyme was added into fresh reaction mixture to catalyze the enzymatic reaction.

### HPLC analysis

For determining the concentrations of 7-ACA and *N*-bromoacetyl-7-ACA, the reaction mixture was analyzed by reversed-phase HPLC on an Eclipse plus C18 column (4.6 mm × 250 mm, 5 μm, Agilent, USA) using a Waters 1525 pump and a 2489 UV detector. The mobile phase was a mixture of acetonitrile and Na_2_HPO_4_-citric acid buffer 1 (20/80, v/v) with a flow rate of 1 mL/min, in which Na_2_HPO_4_-citric acid buffer 1 was composed of 19.4 mmol/L NaH_2_PO_4_ and 6.1 mmol/L citric acid. The UV absorption wavelength was 260 nm. The retention times of 7-ACA and *N*-bromoacetyl-7-ACA were 2.6 and 4.1 min, respectively. For monitoring the acyl donors (methyl bromoacetate and ethyl bromoacetate), the mobile phase was replaced by the mixture of methanol, acetonitrile, and NaH_2_PO_4_-citric acid buffer 2 (40/20/40, v/v), in which Na_2_HPO_4_-citric acid buffer 2 was composed of 6.5 mmol/L NaH_2_PO_4_ and 6.8 mmol/L citric acid. The UV absorption wavelength was 210 nm. The retention times of methyl bromoacetate and ethyl bromoacetate were 3.9 and 4.9 min, respectively.

## Results and discussion

### Enzyme screening

**Fig. 2:**
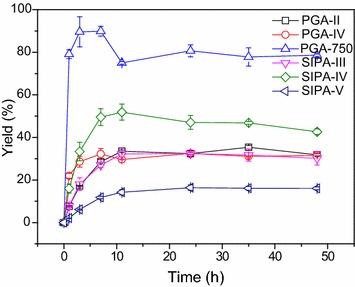
Effect of PA sources on the synthesis of *N*-bromoacetyl-7-ACA. Reaction conditions: 40 mmol/L 7-ACA, 120 mmol/L methyl bromoacetate, 3 U/mL enzyme, 5 mL phosphate buffer (100 mmol/L, pH 7.5), 200 r/min, 20 °C

Initially, six immobilized PAs were examined for kinetically controlled synthesis of *N*-bromoacetyl-7-ACA (Fig. 2), since biocatalyst is a key factor affecting the enzymatic reactions. As shown in Fig. 2, all the enzymes tested could accept methyl bromoacetate as the substrate, and catalyze *N*-bromoacetylation of 7-ACA. However, enzymes from different sources exhibited totally different catalytic performances in the synthesis of *N*-bromoacetyl-7-ACA. As shown in Fig. 2, of the enzymes tested, PGA-750 that gave a yield of approximately 90% after 3 h was the best biocatalyst, while SIPA-V was the worst. No acylation reactions occurred in the absence of enzymes. Previously, Wei and co-workers reported that PA from *B. megaterium* could catalyze efficiently the coupling of d-phenylglycine methyl ester to 7-aminodesacetoxymethyl-3-chlorocephalosporanic acid, thus furnishing cefaclor (Yang and Wei 2003). Therefore, PA from *B. megaterium* may be a versatile biocatalyst which can accept the esters bearing and without the aryl group as the substrates. Except for PGA-750, the maximal yields of less than 52% were obtained with other enzymes after 11 h. In addition, after reaching the maximal yields, further prolongation of reaction time would result in the decreased product yields in all cases, which may be attributed to the occurrence of the side reaction—enzymatic hydrolysis of the desirable product *N*-bromoacetyl-7-ACA. In addition to the product yields, the 7-ACA conversions were monitored (Additional file 1: Figure S1). It could be found that the substrate conversions increased remarkably as the reaction proceeded in all cases; besides, the substrate conversions were higher than the corresponding yields. The stability of 7-ACA in the absence and presence of the immobilized enzyme was tested (Additional file 1: Figure S2). It was found that 7-ACA was very stable in buffer without enzyme, while it was quickly degraded into two unknown compounds within 4 h in the presence of enzyme. Hence, the reason for the fact that the conversions were generally higher than the yields might be attributed to the significant enzymatic hydrolysis of 7-ACA. It was reported that cephalosporin-C deacetylase and acetyl xylan esterase were capable of hydrolyzing 7-ACA into its deacetylated derivative (Takimoto et al. 2004; Montoro-García et al. 2010). Presently, the enzyme(s) responsible for the 7-ACA decomposition remains unknown, which is underway in our laboratory.

### Effect of acyl donors

**Fig. 3:**
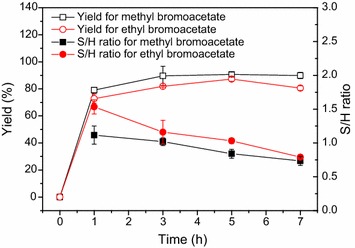
Effect of acyl donors on the synthesis of *N*-bromoacetyl-7-ACA. Reaction conditions: 40 mmol/L 7-ACA, 120 mmol/L bromoacetate, 3 U/mL PGA-750, 10 mL phosphate buffer (100 mmol/L, pH 7.5), 200 r/min, 20 °C

Figure 3 shows the effect of acyl donors on the synthesis of *N*-bromoacetyl-7-ACA. It was found that the reaction rate appeared to be slightly higher with methyl bromoacetate as the acyl donor than that with ethyl ester; additionally, a slightly higher product yield (approximately 90%) was obtained with the former. Besides, the substrate conversions were more than 95% after 7 h. It suggests that the two esters tested are good acyl donors for the enzymatic *N*-acylation of 7-ACA. In addition to the conversions and yields, the S/H ratios (the molar ratios of *N*-bromoacetyl-7-ACA to bromoacetic acid, the selectivity toward synthesis), a parameter that is often used to assess the economics of the enzymatic process (Bruggink et al. 1998; Wegman et al. 2002; Ribeiro et al. 2005; Cao et al. 2000), were also tracked (Fig. 3). As shown in Fig. 3, the S/H ratios were higher with ethyl ester as the acyl donor than those with methyl ester. Furthermore, the S/H ratios decreased significantly as the reaction progressed in the two cases, which is consistent with the previous results (Cao et al. 2000). The S/H ratios were approximately 1.0 with both methyl and ethyl esters, when the maximal yields were achieved. In terms of the yield, reaction rate, and S/H ratio, methyl bromoacetate was considered as the optimal acyl donor and used in the following studies.

### Effect of key conditions

**Fig. 4:**
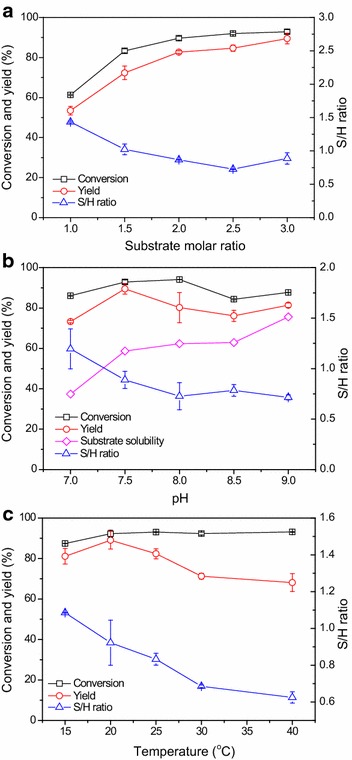
Effect of substrate molar ratio (**a**), pH (**b**), and temperature (**c**) on the synthesis of *N*-bromoacetyl-7-ACA. Reaction conditions for part a: 40 mmol/L 7-ACA, methyl bromoacetate of the designated concentration, 3 U/mL PGA-750, 10 mL phosphate buffer (100 mmol/L, pH 7.5), 200 r/min, 20 °C; reaction conditions for part b: 40 mmol/L 7-ACA, 120 mmol/L methyl bromoacetate, 3 U/mL PGA-750, 10 mL buffer (100 mmol/L, pH 7.0–8.0, phosphate buffer; pH 8.5–9.0, Tris–HCl buffer), 200 r/min, 20 °C; reaction conditions for part c: 40 mmol/L 7-ACA, 120 mmol/L methyl bromoacetate, 3 U/mL PGA-750, 10 mL phosphate buffer (100 mmol/L, pH 7.5), 200 r/min, the designated temperature

Some key conditions including the substrate molar ratio (the molar ratio of methyl bromoacetate to 7-ACA), pH, and temperature were optimized to improve the enzymatic synthesis of *N*-bromoacetyl-7-ACA (Fig. 4). Figure 4a shows the influence of the substrate molar ratio on the enzymatic reaction when the concentration of 7-ACA was 40 mmol/L. As shown in Fig. 4a, increasing the substrate molar ratio resulted in the significant improvement in the conversions as well as the yields. For example, the maximal conversion and yield were 61% and 53%, respectively, with the substrate molar ratio of 1.0, while being 90% and 83%, respectively, with the substrate molar ratio of 2.0. Nonetheless, using lower substrate molar ratios seemed to be favorable for synthesis, resulting in the higher S/H ratio, which is in good agreement with the previous results (Ferreira et al. 2007; Ribeiro et al. 2005).

The effect of pH on the enzymatic reaction and the 7-ACA solubility was studied, when pH varied from 7.0 to 9.0 (Fig. 4b). As shown in Fig. 4b, pH had a significant effect on the product yield. For example, the product yield was approximately 73% at pH 7.0, while it increased to 89% at pH 7.5. The highest product yield was observed at pH 7.5. It has been well demonstrated that alkaline pH can facilitate the enzymatic hydrolytic reactions (Ospina et al. 1996), which may explain the results obtained in this work that the yields (76–81%) are lower under higher pH. As shown in Fig. 4b, the S/H ratio decreased markedly from 1.2 to 0.7 when pH increased from 7.0 to 8.0, suggesting that PA catalyzed preferably the hydrolytic reaction under alkaline pH. Nonetheless, higher solubility of 7-ACA was found in alkaline media, likely due to the presence of the carboxylic acid group in this molecule. The 7-ACA solubility reached approximately 76 mmol/L in pH 9.0 Tris–HCl buffer (100 mmol/L), and its solubility was closely dependent on the buffer types (Additional file 1: Figure S3). To identify the optimal pH for this enzyme reaction, the stability of 7-ACA and the immobilized enzyme were also studied under various pH (Additional file 1: Figures S4 and S5). As shown in Additional file 1: Figure S4, the changes in the 7-ACA concentrations appeared not to be correlated with pH, and 7-ACA of about 10 and 25% degraded after 3 and 24 h in all cases, respectively. In addition, the immobilized enzyme PGA-750 appeared to be very stable at various pH (Additional file 1: Figure S5). In particular, the enzyme almost retained its original activity after incubation of 216 h at pH 7.5 and 9.0, which may be partially accounted for by the fact that the protein was covalently immobilized on the carrier. The excellent stability of this immobilized enzyme might highlight its industrial application potential.

The effect of reaction temperature on the synthesis of *N*-bromoacetyl-7-ACA is shown in Fig. 4c. It was found that reaction temperature exerted a substantial effect on the product yield. The highest product yield was obtained at 20 °C. Higher temperature resulted in the lower yields when the temperature was more than 20 °C, which is in good agreement with previous results in the enzymatic synthesis of other semi-synthetic antibiotics (Aguirre et al. 2006; Wei et al. 2003). The reason might be that, as compared to synthesis, the hydrolytic reactions became predominant at high temperature (e.g., 30 and 40 °C), which could be verified by the lower S/H ratios at higher temperature (Fig. 4c).

### Effect of the substrate concentrations

**Fig. 5:**
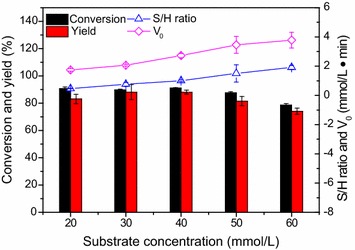
Effect of the substrate concentrations on the synthesis of *N*-bromoacetyl-7-ACA. Reaction conditions: 7-ACA of the designated concentration, substrate molar ratio (methyl bromoacetate/7-ACA) of 3, 3 U/mL PGA-750, 10 mL phosphate buffer (100 mmol/L, pH 7.5), 200 r/min, 20 °C

The effect of the 7-ACA concentrations on the enzymatic reaction is exhibited in Fig. 5. The substrate conversions (approximately 90%) as well as the product yields (83–88%) were found to be comparable when the substrate concentrations were less than 40 mmol/L. However, the conversion and yield decreased significantly to 79 and 74%, respectively, with the substrate concentration of 60 mmol/L. In addition, the initial reaction rate increased with increasing the substrate concentration, suggesting that there exists no substrate inhibition within the concentration range tested. Interestingly, it was found that the S/H ratio appeared to be higher at higher substrate concentration, and the S/H ratios of more than 1.5 were achieved when the 7-ACA concentrations were beyond 50 mmol/L. Previously, other groups also demonstrated that the reactions preferred synthesis to hydrolysis at higher reactant concentrations (Youshko et al. 2000, 2004). The substrate concentration of 50 mmol/L was used in the subsequent studies, due to the good S/H ratio and satisfactory product yield.

### Effect of enzyme dosage

**Fig. 6:**
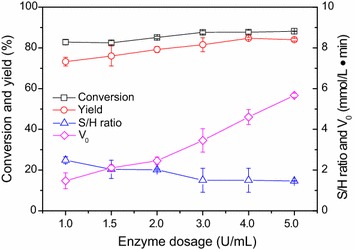
Effect of enzyme dosage on the synthesis of *N*-bromoacetyl-7-ACA. Reaction conditions: 50 mmol/L 7-ACA, 150 mmol/L methyl bromoacetate, PGA-750 of the designated dosage, 10 mL phosphate buffer (100 mmol/L, pH 7.5), 200 r/min, 20 °C

The optimization of enzyme dosage was conducted to improve the enzymatic reaction (Fig. 6). As shown in Fig. 6, the initial reaction rate increased significantly with the increment of enzyme dosage. For instance, the initial reaction rate was around 1.5 mmol/L min with the enzyme dosage of 1.0 U/mL, while it increased substantially to 5.7 mmol/L min with 5.0 U/mL. In addition, the increase in the enzyme dosages led to the improved yields, and the highest yield of up to 85% was achieved with the enzyme dosage of 4 U/mL. The optimal enzyme dosage was regarded as 4 U/mL, where the S/H was around 1.5.

### Operational stability of the immobilized enzyme

**Fig. 7:**
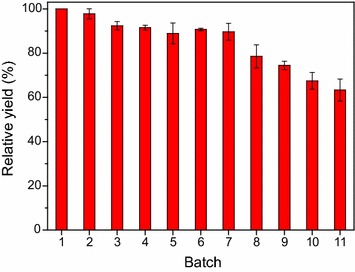
Operational stability of the immobilized enzyme PGA-750. Reaction conditions: 50 mmol/L 7-ACA, 150 mmol/L methyl bromoacetate, 4 U/mL PGA-750, 10 mL phosphate buffer (100 mmol/L, pH 7.5), 200 r/min, 20 °C. The reaction time of each batch was 2 h. The relative yield of the first batch was defined as 100%

Finally, the operational stability of the immobilized enzyme PGA-750 was studied under the optimal reaction conditions (Fig. 7). It could be found that the immobilized enzyme had good stability, which is in good agreement with the above results (Additional file 1: Figure S5). No significant deactivation was observed during 7 runs, and the relative yield of approximately 90% was obtained in 7th batch. Unfortunately, the immobilized enzyme significantly lost its activity in the following batches. The relative yield decreased to around 63% at 11th batch. As described above, bromoacetic acid and methanol would be produced as the by-products during the enzymatic synthesis of the desired product (Scheme 1), which may have a detrimental effect on the enzyme stability.

## Conclusions

An enzymatic approach to *N*-bromoacetyl-7-ACA, the key intermediate for the industrial production of cefathiamidine, has been developed successfully in a fully aqueous medium in this work. Apparently, this new enzymatic process is superior to traditional chemical methods, because of being simple, mild reaction condition, producing less waste, and being environmentally benign. More importantly, the yield of the desired product in this enzymatic process is much higher than that in the traditional chemical processes (85 vs 73%). Therefore, the enzymatic method exhibited promising application potential for the chemoenzymatic production of cefathiamidine. However, many problems associated with this enzymatic process such as relatively low substrate concentrations and unsatisfactory operational stability for its large-scale application may be addressed by the corresponding measures (e.g., using the supersaturated substrate solutions and fed-batch feeding strategy, and improving enzyme immobilization methods) (Volpato et al. 2010; Ferreira et al. 2007; Youshko et al. 2004; Illanes et al. 2007; Montes et al. 2007).

## Additional file

**Additional file 1.** Additional figures including: **Figure S1.** Time courses of 7-ACA transformation catalyzed by various immobilized enzymes; **Figure S2.** Stability of 7-ACA in the absence and presence of PGA-750; **Figure S3.** The 7-ACA solubility in various buffer; **Figure S4.** Stability of 7-ACA at different pH; **Figure S5.** The stability of the immobilized enzyme PGA-750 at different pH.
