# Supplementary material for: Penicillin acylase-catalyzed synthesis of N-bromoacetyl-7-aminocephalosporanic acid, the key intermediate for the production of cefathiamidine
Source: Bioresour Bioprocess. 2016 Nov 19;3(1):49. doi: 10.1186/s40643-016-0127-3 (PMC5116309; doi:10.1186/s40643-016-0127-3)
Supplement: Supplementary file 1 — Additional file 1. Additional figures including: Figure S1. Time courses of 7-ACA transformation catalyzed by various immobilized enzymes; Figure S2. Stability of 7-ACA in the absence and presence of PGA-750; Figure S3. The 7-ACA solubility in various buffer; Figure S4. Stability of 7-ACA at different pH; Figure S5. The stability of the immobilized enzyme PGA-750 at different pH. [file 40643_2016_127_MOESM1_ESM.pdf]

Supporting Information

Penicillin acylase-catalyzed synthesis of *N*-bromoacetyl-7-aminocephalosporanic acid, the key intermediate for the production of cefathiamidine

Xiao-Li Zhang, Min-Hua Zong, Ning Li*

School of Food Science and Engineering, South China University of Technology, 381 Wushan Road, Guangzhou 510640, China

*Correspondence: lining@scut.edu.cn

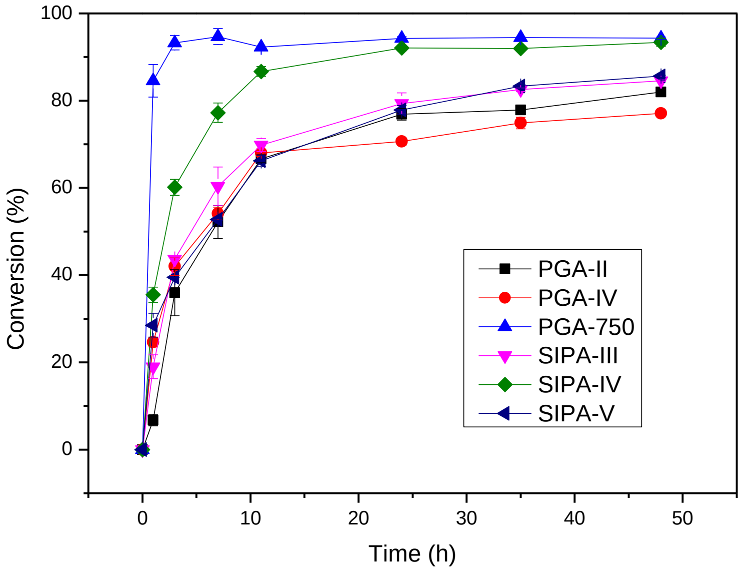

Fig. S1 Time courses of 7-ACA transformation catalyzed by various immobilized enzymes

Reaction conditions: 40 mmol/L 7-ACA,120 mmol/L methyl bromoacetate, 3 U/mL enzyme, 5 mL phosphate buffer (100 mmol/L, pH 7.5), 200 r/min, 20 °C

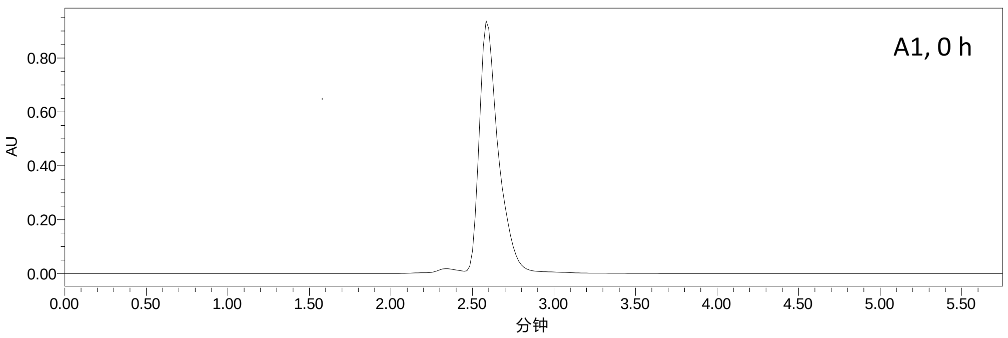
A1, 0 h
AU
分钟

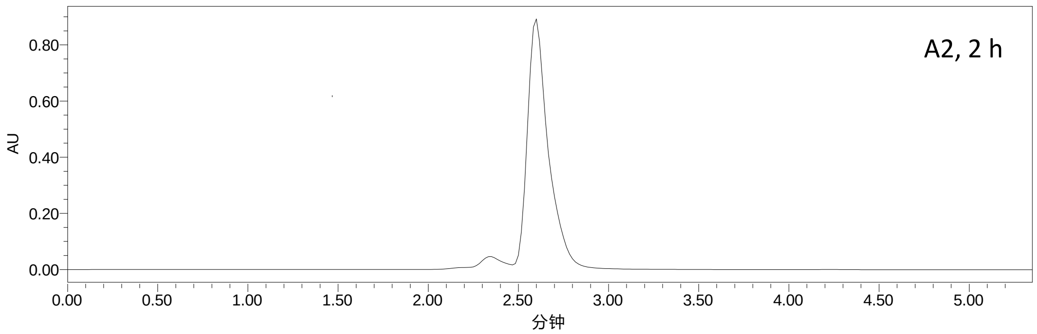
A2, 2 h
AU
分钟

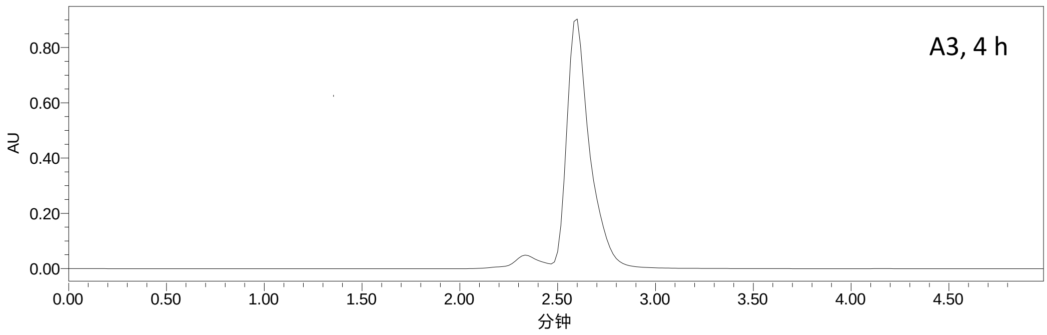
A3, 4 h
AU
分钟

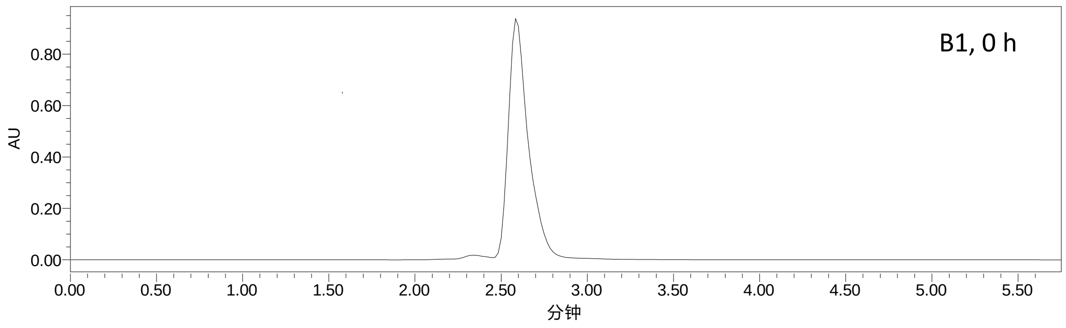
B1, 0 h
AU
分钟

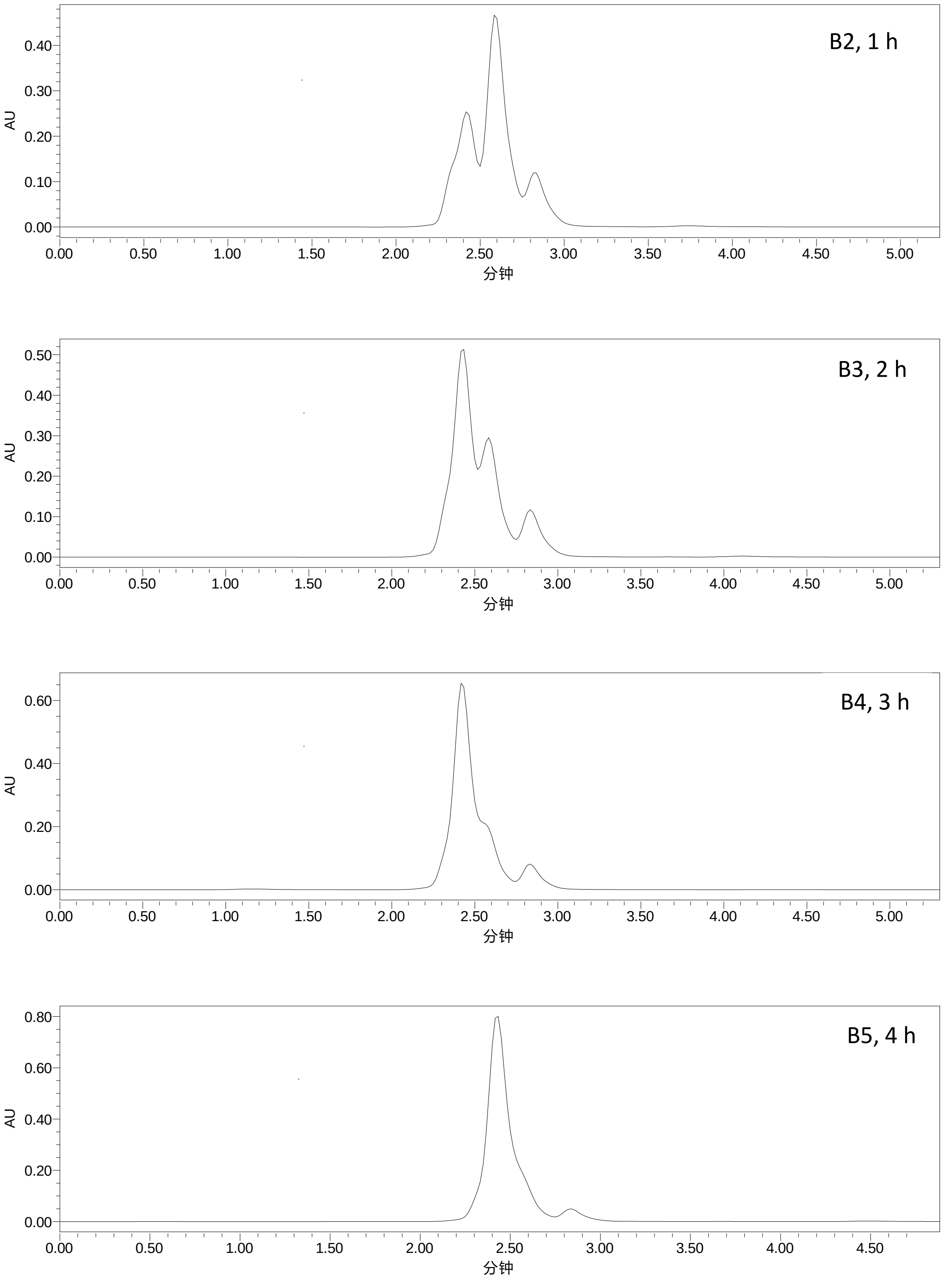

Fig. S2 Stability of 7-ACA in the absence and presence of PGA-750

Conditions: 40 mmol/L 7-ACA, 10 mL phosphate buffer (100 mmol/L, pH 7.5), in the absence (A) or presence (B) of 3 U/mL PGA-750, 20 °C, 200 r/min

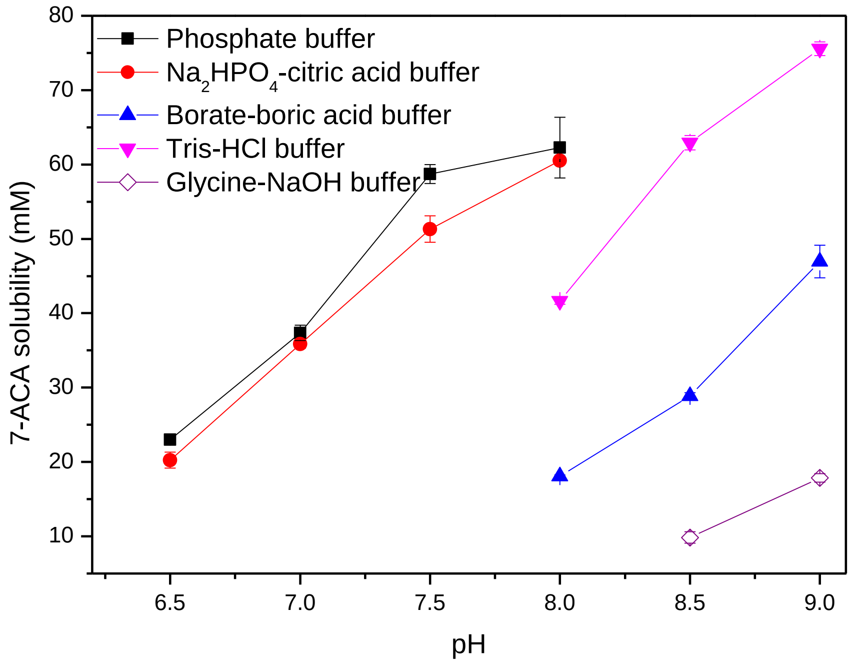

Fig. S3 The 7-ACA solubility in various buffer

Conditions: Excess 7-ACA was added into various buffer (100 mmol/L), and incubated at 20 °C and 200 r/min for 2 h; then, the supernatant was withdrawn for HPLC analysis.

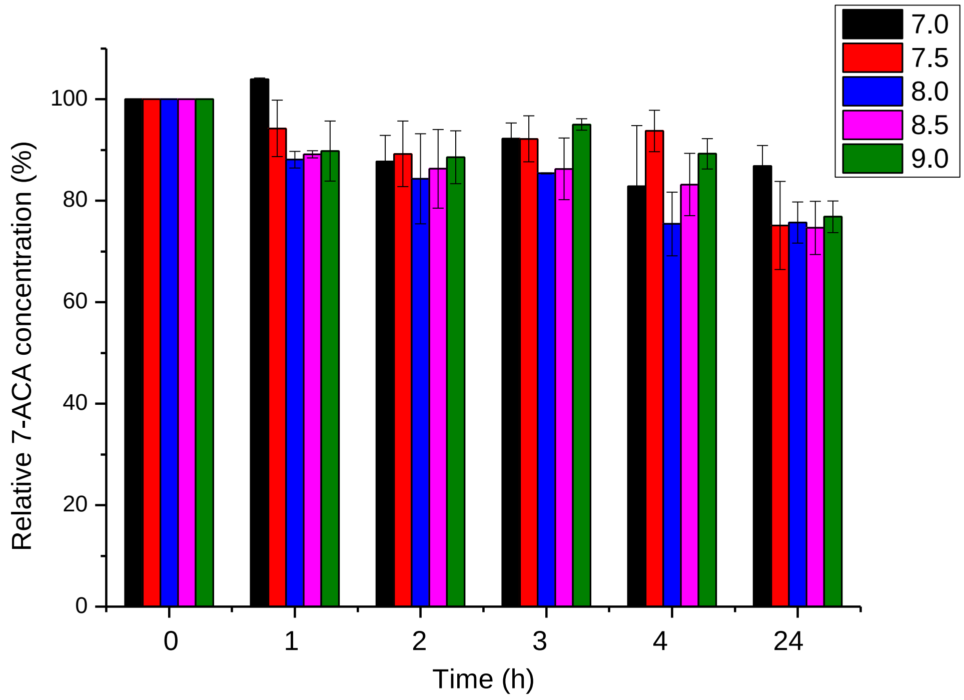

Fig. S4 Stability of 7-ACA at different pH

Conditions: 30 mmol/L 7-ACA, 20 °C, 200 r/min, 5 mL buffer (100 mmol/L, different

pH)

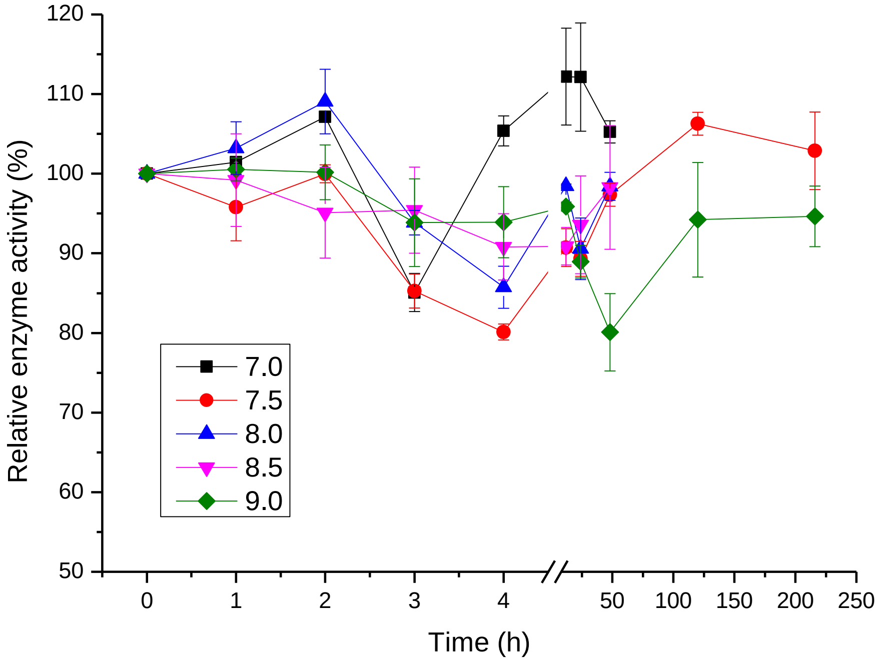

Fig. S5 The stability of the immobilized enzyme PGA-750 at different pH Conditions: The immobilized enzyme was incubated at 20 °C and 200 r/min at different pH (7.0-8.0, 100 mmol/L phosphate buffer; 8.5 and 9.0, 100 mmol/L Tris-HCl buffer). The enzyme was isolated at specified time intervals, and its activity was determined spectrophotometrically using *p*-dimethylaminobenzaldehyde as the substrate [1]. The relative enzyme activity of the native enzyme was defined as 100%.

References

[1] Shewale JG, Kumar KK, Ambekar GR. Evaluation of determination of 6-aminopenicillanic acid by *p*-dimethyl aminobenzaldedyde. Biotechnol Tech. 1987;1:69-72.
